# Bioactivity of Thyroid Hormone Analogs at Cancer Cells

**DOI:** 10.3389/fendo.2018.00739

**Published:** 2018-12-04

**Authors:** Paul J. Davis, Heng-Yuan Tang, Aleck Hercbergs, Hung-Yun Lin, Kelly A. Keating, Shaker A. Mousa

**Affiliations:** ^1^Department of Medicine, Albany Medical College, Albany, NY, United States; ^2^Pharmaceutical Research Institute, Albany College of Pharmacy and Health Sciences, Rensselaer, NY, United States; ^3^Department of Radiation Oncology, The Cleveland Clinic, Cleveland, OH, United States; ^4^PhD Program for Cancer Molecular Biology and Drug Discovery, College of Medical Science and Technology, Taipei Medical University, Taipei, Taiwan; ^5^Taipei Cancer Center, Taipei Medical University, Taipei, Taiwan; ^6^Traditional Herbal Medicine Research Center of Taipei Medical University Hospital, Taipei Medical University, Taipei, Taiwan; ^7^TMU Research Center of Cancer Translational Medicine, Taipei Medical University, Taipei, Taiwan

**Keywords:** L-thyroxine, tetrac, triac, reverse T3, non-genomic actions

## Abstract

In the context of genomic thyroid hormone actions in normal (noncancer) cells that involve primary interactions with nuclear thyroid hormone receptors (TRs), L-thyroxine (T4), and 3,3′,5′-triiodo-L-thyronine (reverse T3, rT3) have little bioactivity. In terms of TRs, T4 is a prohormone from which the active nuclear ligand, 3,5,3′-triido-L-thyronine (T3), is generated by deiodination. Deaminated T4 and T3 metabolites have different genomic effects: tetraiodothyroacetic acid (tetrac) is a low grade thyromimetic derivative of T4, whereas triiodothyroacetic acid (triac), the acetic acid metabolite of T3, has substantial thyromimetic activity. In cancer cells, the cell surface receptor for thyroid hormone on integrin αvβ3 mediates non-genomic actions of thyroid hormone analogs. The integrin is expressed in large measure by cancer cells and dividing endothelial cells and has a substantially different panel of responses to thyroid hormone analogs. At αvβ3, T4 is a potent proliferative, anti-apoptotic and pro-angiogenic hormone and is the primary ligand. rT3 may also be proliferative at this site. In contrast, tetrac and triac are antagonists of T4 at αvβ3, but also have anticancer properties at this site that are independent of their effects on the binding of T4.

## Introduction

The concept that the bioactivity of thyroid hormone is expressed by 3,5,3′-triiodo-L-thyronine (T3) has served to identify the critical metabolic and protein synthetic functions of the hormone that are dependent upon interactions of T3 with nuclear thyroid hormone receptors (TRs) in normal cells (1, 2). The T3-TR mechanism of action of thyroid hormone is designated genomic. T3 also has a limited number of effects initiated in cytoplasm or at the plasma membrane that are independent of TRs and thus are non-genomic in mechanism (1). T3 has also been recognized to have specific functions in cancer cells that may depend upon TR mutation (3, 4). The primary thyroid hormone product of the thyroid gland is L-thyroxine (T4), whose status as a prohormone for T3 has been fully appreciated to be defined by deiodinases in a variety of non-thyroid tissues that generate T3 by outer thyroid hormone ring deiodination at the 5' position of the diphenyl ether structure of iodothyronines (5). Inner ring deiodination at the 5 position produces reverse T3 (rT3), which is generally regarded as inactive. Modifications of the alanine side chain of iodothyronines occur naturally at the cellular level, but only to a limited degree, and yields tetraiodothyroacetic acid (tetrac) and triiodothyroacetic acid (triac), which have some metabolic activities (6).

The appreciation of the existence of a receptor for thyroid hormone analogs on the plasma membrane of cancer and rapidly dividing endothelial cells (1, 7, 8) has enabled the recognition of functions of thyroid hormone analogs that were previously thought to be inactive. The cell surface receptor is on the extracellular domain of integrin αvβ3. The plasma membrane receptor for thyroid hormone has no structural homologies with TRs. At this receptor, T4 promotes cancer cell proliferation, supports anti-apoptosis and enhances angiogenesis because of its presence on endothelial cells (9–11). Tetrac inhibits the actions of T4 at the integrin and in the absence of T4 has a variety of actions on expression of specific genes. That is, T4 and tetrac affect the activities of a number of signal transduction pathways that downstream modulate differentially the transcription of a number of genes (8, 12, 13). The actions of T4 and tetrac at αvβ3 are non-genomic in that they do not directly involve TRs or require hormonal presence in the nucleus. Operationally, however, both genomic and non-genomic actions of thyroid hormones may culminate in specific gene transcription. The receptor on αvβ3 can also control the trafficking of intracellular proteins—including the transfer of cytoplasmic TRs and estrogen receptor-α into the nuclear compartment of cancer cells—and regulate the phosphorylation/activation of TRs (1, 14).

Integrins are a family of two dozen heterodimeric structural proteins of the plasma membrane and are critical to tissue structure and to cell migration. They interact importantly with extracellular matrix proteins and with other cells (15). The receptor for thyroid hormone on αvβ3 is the first small molecule binding site recognized on integrins, but subsequently discrete receptors on αvβ3 have been reported for other small molecules, including dihydrotestosterone (DHT) (16) and the stilbene, resveratrol (17). Integrin αvβ3 is expressed to a limited degree by non-dividing normal, i.e., non-cancer, cells, but the function of such cells does not appear to be affected by any interactions of T4 and the integrin that may occur. This may reflect the (“non-activated”) physical state of αvβ3 in non-cancer cells (18). The integrin is functional, however, on the surface of platelets, reflecting the presence of fractions of the plasma membrane of megakaryocytes. T4 has been shown to induce platelet aggregation via αvβ3 (19).

In the succeeding sections, we briefly review the bioactivities of thyroid hormone analogs at the iodothyronine receptor on integrin αvβ3.

## Bioactivity of T4 at αvβ3

An early demonstration of the non-genomic activity of T4 was its conversion of soluble actin to fibrous actin F-actin (8, 20). This was initially demonstrated in astrocytes and glial cells. The molecular basis of this action of the hormone is incompletely understood, but appears to involve a truncated TRα (TRΔα1) isoform in cytoplasm (8). This isoform does not contain a nuclear localization signal. T3 does not affect the state of actin in cells. Regulation of the state of actin is of obvious importance to both normal and malignant cells.

In 2004, T4 was shown to be pro-angiogenic in the chick choriollantoic membrane (CAM) model. The CAM model has important dependency on αvβ3, and antibody to this integrin blocked the action of T4 on new blood vessel formation. Physiological concentrations of free T4 were shown to be active in this system (21). In this model system, the inhibition of conversion of T4 to T3 did not affect the action of T4 on the state of actin. This action of T4 was shown to be initiated at integrin αvβ3, leading to a series of studies characterizing the receptor on the head of the extracellular domain of the integrin. While integrins are found on both normal cells and malignant cells, αvβ3 is particularly generously expressed by cancer cells and rapidly dividing endothelial cells (8, 15). It and other integrins are very important to cell-cell interactions and cell-extracellular matrix (ECM) proteins that underlie tissue integrity and the orientation of motile cells. As noted above, thyroxine was the first small molecule to be found to bind specifically to αvβ3.

At the integrin, T4 was found to have a number of cancer-relevant functions mediated by αvβ3 (1, 8, 9, 12). These included stimulation of cell proliferation and anti-apoptosis (10). Such properties relied upon activation of signaling pathways [mitogen activated protein kinase [MAPK]/ERK1/2; phosphatidylinositol 3-kinase (PI3K)] that culminated downstream in specific gene transcription (1, 7, 8, 12). In contrast to the genomic actions of T3 that depend on primary intranuclear interactions of T3 with activated TRs, the actions of T4 on cancer cell proliferation, angiogenesis and apoptosis depended on the location of the hormone on the cell surface (1, 8).

Among the genes whose expression is differentially regulated from the plasma membrane and αvβ3 are genes for matrix metalloproteinases, basic fibroblast growth factor (FGF2), hypoxia-inducible factor-1α (HIF-1α), cyclooxygenase-2 (COX-2) and, interestingly, TRα and TRβ; transcription of all of the preceding genes is upregulated by T4 (8). The control of TR gene expression from the cell surface is an example of overlapping non-genomic and genomic effects of thyroid hormone (14). Downregulated is expression of the genes for pro-apoptotic APAF1, CASP3, PMAIP1, and BBC3 (8). The relevance of these genes to cancer cell survival is clear. Tetrac is a naturally occurring derivative of T4 that blocks the binding of T4 (and T3) to the receptor on αvβ3. In the absence of T4, however, tetrac and tetrac that is modified by covalent binding to a nanoparticle or polymer have effects on expression of several hundred genes (12), e.g., angiogenesis-linked vascular endothelial growth factor A (VEGFA), epidermal growth factor receptor (EGFR), cell survival pathway genes *XIAP* and *MCL1* and cell cycle-regulating genes. The latter include genes for multiple cyclins and a cyclin-dependent kinase. Genes relevant to radioresistance and chemoresistance, e.g., p-glycoprotein (*P-gp*) are also affected by tetrac. The expression of all of these genes is decreased by unmodified or modified tetrac, indicating that the modified tetrac compounds have applications as experimental chemotherapeutic agents. Because tetrac is an antagonist of T4, a possible implication of these studies of tetrac is that T4 may be a stimulator of the transcription of this panel of genes. This possibility has not yet been systematically examined.

## Bioactivity of rT3

The conversion of T4 to rT3, rather than to T3, generates a thyroid hormone analog with no genomic actions. This 5-deiodination process is a function of the action of deiodinase 3 (DIO3, D3) or deiodinase 1 (DIO1, D1) (6). rT3, however, was found a number of years ago to be capable of converting soluble actin to F-actin (20), just as T4 does. In developing mouse cerebellum, astrocytes lacking TRs recover normal actin function with transfection of TRΔα1 (22).

rT3 may also modulate avian lipid metabolism response to epinephrine and steroids (23). The activity of DIO2 (D2) in murine neuroblastoma cells may also be reduced by rT3 (24). Thus, a set of observations in disparate model systems indicates that rT3 has bioactivity. Against this background, we have recently tested rT3 for proliferative activity in glioblastoma cells. We had previously shown that T4 enhances proliferation of several glioma cell lines (25) and that chemically modified tetrac, a T4 antagonist, inhibited the growth of glioblastoma xenografts (26). In the recent studies, the glioblastoma cell line U87MG and two primary cultures of human GBM cells significantly increased their rates of proliferation *in vitro* when exposed to T4, as expected, but also to rT3 (27). These studies must be extended and expanded to include other types of cancer. Confirmation would indicate that conversion of T4 to rT3, rather than to T3, offers cancer cells another thyroid hormone analog support mechanism. Indeed, T3 at physiological concentrations may provide no stimulus to tumor cell proliferation, as a recent clinical study in endstage cancer patients of euthyroid hypothyroxinemia suggests (28). In that study, stabilization or regression of advanced disease was achieved with inhibition of endogenous thyroid hormone production by methimazole and maintenance of the euthyroid state with exogenous T3. Elimination of host T4 production in such patients also minimizes production of rT3.

We can conclude that rT3 has bioactivity and that, possibly, this thyroid hormone analog has proliferative activity on certain cancer cells.

## Tetrac and Triiodothyroacetic Acid (Triac)

In the nucleus, tetrac and triac are thyromimetic (6). Triac has some TRβ-selectivity that has favored its use over tetrac in thyroid hormone-resistant patients to suppress host thyrotropin (TSH) (6), but each agent has been used in this setting. Advantages of the genomic effects of these deaminated derivates of T4 and T3 have also been sought in management of obesity and hyperlipidemia. All such applications involve hormone effects on non-cancer cells.

Because of the heightened expression of αvβ3 in cancer cells, non-genomic actions of tetrac and triac are seen in such cells. Both are anti-proliferative in cancer cells (8). Tetrac has been chemically modified to a nanoparticulate drug (Nanotetrac, NDAT) by covalent coupling to large molecules such as poly-lactic-co-glycolic acid (PLGA) to minimize its access to the intranuclear compartment when the agent is internalized by cells. Tetrac is thyromimetic in the intranuclear compartment (29). Chemically modified tetrac blocks binding of T4 (and T3) to the thyroid hormone receptor on αvβ3, thus eliminating some of the cancer support properties of T4 that were described above. In addition, in the absence of T4, NDAT or tetrac in another formulation in our laboratory in which it is covalently bound to polyethylene glycol (PEG) has actions downstream of the integrin on expression of a large number of cancer-relevant genes (8, 12, 13). The actions are anti-proliferative, pro-apoptotic and anti-angiogenic by multiple mechanisms. Modified tetrac may also impair DNA repair that is important to cancer cell resistance to radiation (30). Finally, by suppressing expression of the *P-gp* gene, modified tetrac may reduce chemoresistance (31), since the plasma membrane P-gp pump exports certain cancer chemotherapeutic drugs (31, 32).

X-irradiation has been shown to activate integrin αvβ3 (18), an effect that is primarily on the β3 monomer and that is thought to contribute to radioresistance (33). This effect is blocked by tetrac (as NDAT).

The actions of triac on cancer cells have been incompletely characterized. It is clear, however, that triac can act at integrin αvβ3 to non-genomically initiate apoptosis in human ovarian cancer cells (34). Triac does not appear to have effects on mitochondria in tumor cells (35). How important genomic effects of triac may be in cancers cells is not known. Triac not surprisingly binds to a genetically modified TRβ that trafficks between cytoplasm and the nucleus in a cancer cell model generated to detect endocrine disrupting chemicals (36), and triac binds to TR in non-cancer cells (37) and has been used clinically to treat certain forms of thyroid hormone resistance because of its facilitated transport across the plasma membrane of normal cells.

It should also be noted that thyroid hormone analogs affect abundance of certain microRNAs (mRNAs) in cancer cells. We have pointed out that Nanotetrac differentially regulates transcription of miR-21 and miR-15A in tumor cells (12, 38) and in so doing is anti-angiogenic and decreases metastatic potential of cancer cells. The effects of T4 on expression of these miRNAs has not yet been examined.

What is apparent is that the thyromimetic properties of deaminated thyroid hormone, particularly tetrac, are importantly overshadowed in cancer cells by its anticancer actions that are expressed via integrin αvβ3. Triac also has anticancer activity at the integrin.

## Conclusions

The existence of integrin αvβ3 on the surface of cancer cells and rapidly dividing endothelial cells provides new insights into the bioactivities of a number of iodothyronines. T4 is a potent pro-angiogenic hormone with proliferative-enhancing activity in cancer cells, whereas in the context of nuclear TRs, T4 is simply a prohormone for T3. At the thyroid hormone receptor on αvβ3, rT3 may also be biologically active. Finally, deaminated thyroid hormone analogs are weak thyromimetic agents at TRs, but are potent anti-T4 agents in cancer cells and also have a number of anticancer effects that are independent of their activity as blockers of T4 binding to the cell surface receptor.

What about T3 at αvβ3? In higher than physiologic concentrations, T3 can stimulate cancer cell proliferation at the receptor on the integrin (8, 39), but as the induced clinical state of euthyroid hypothyroxinemia in cancer patients suggests, maintenance of euthyroidism exclusively with T3 does not appear to promote tumor growth (28).

The scope of this review is limited by the relatively small number of types of solid tumors that have been studied for possible effects of thyroid hormone analogs. Leukemic cells have not been examined for trophic or antagonistic effects of thyroid hormones. Additional clinical studies are needed of the effects of induction of euthyroid hypothyroxinemia on tumor behavior.

An inference of the discussion of tetrac is the need to test clinically the anticancer efficacy of chemically modified tetrac that has been demonstrated experimentally in cells and in xenografts.

## Author Contributions

All authors listed have made a substantial, direct and intellectual contribution to the work, and approved it for publication.

### Conflict of Interest Statement

Coauthors PJD and SAM have stock holdings in a company that is developing modified tetrac as an anticancer agent and author KAK is a paid consultant of NanoPharmaceuticals LLC. The remaining authors declare that the research was conducted in the absence of any commercial or financial relationships that could be construed as a potential conflict of interest.
